# *In Situ* Characterization of Follicular Helper CD4 T Cells Using Multiplexed Imaging

**DOI:** 10.3389/fimmu.2020.607626

**Published:** 2021-02-03

**Authors:** Kalliopi Ioannidou, Daba-Rokhya Ndiaye, Alessandra Noto, Craig Fenwick, Sotirios P. Fortis, Giuseppe Pantaleo, Constantinos Petrovas, Laurence de Leval

**Affiliations:** ^1^Department of Laboratory Medicine and Pathology, Institute of Pathology, Lausanne University Hospital and Lausanne University, Lausanne, Switzerland; ^2^Service of Immunology and Allergy, Department of Medicine, Lausanne University Hospital, University of Lausanne, Lausanne, Switzerland; ^3^Tissue Analysis Core, Vaccine Research Center, National Institute of Allergy and Infectious Diseases (NIAID), National Institutes of Health (NIH), Bethesda, MD, United States

**Keywords:** follicular helper T cells, multiplexed imaging, heterogeneity, germinal center, tonsil, angioimmunoblastic T-cell lymphoma

## Abstract

Follicular helper CD4 T (Tfh) cells play an essential role in the formation of germinal centers (GCs), where mature B cells proliferate, differentiate, and provide long-term protective humoral responses. Despite the extensive phenotypic characterization and identification of human Tfh cell subsets, their spatial positioning at tissue level is not well understood. Here, we describe a quantitative multiplexed immunofluorescence approach allowing for the comprehensive *in situ* characterization of Tfh cells in human tonsils and lymph nodes (LNs) from individuals with angioimmunoblastic T-cell lymphoma (AITL). We have developed eight multiplexed panels comprising a spectrum of Tfh cell markers, like PD-1, CXCR5, and ICOS, along with transcription factors (Bcl6, Tbet, GATA3), to assess their expression, frequencies, spatial distribution and co-localization in a quantitative manner. Combined analysis of relevant markers revealed the presence of several Tfh cell subsets at tissue level based on the differential expression of surface receptors, nuclear factors as well as their distinct localization within the follicular areas. Interestingly, we found a considerable amount of tonsillar Tfh cells expressing high levels of the Th2 regulator GATA3. The co-expression of GATA3, CXCR5, and BCL6, points to an important role of GATA3 for the generation of effector human Tfh cells. Furthermore, our data revealed significantly different Tfh cell profile signatures between health and disease. Therefore, our imaging platform generates meaningful information for the *in situ* characterization of human Tfh cells and could provide the base for future studies aiming to a comprehensive understanding of Tfh cell tissue heterogeneity.

## Introduction

Tfh cells is a highly specialized subset of CD4+ T cells playing a critical role in protective immunity by helping B cells to produce antibodies against foreign pathogens. They are located in secondary lymphoid organs (SLOs), including tonsil, spleen and lymph nodes. These organs accommodate numerous lymphocytes, organized in T- and B-cell zones with Tfh cells found exclusively in the B-cell zone (1). Therefore, Tfh cells are essential for the formation of a GC reaction and they provide developmental cues for the differentiation of antigen-selected high-affinity GC B cells into memory B cells and plasma cells (2, 3) Tfh cells express unique i) phenotype, characterized by CCR7^lo^, CXCR5^hi^, PD-1^hi^, CTLA-4^hi^, ICOS^hi^, CD95hi, CD40Lhi, and Bcl-6^hi^ expression (4–6) which is preserved across different species (mouse, human, and non-human) (7–10), ii) transcriptome signature (10–12) and iii) function, mediated by both surface receptor interactions and soluble factors like IL-21, IL-4, CXCL13 (3, 10, 13, 14). Within the germinal center Light Zone (LZ), the interaction between Tfh cells, B cells, follicular dendritic cells (FDC) and antigen provides appropriate signals for further division of B cells and their somatic hypermutation in the Dark Zone (DZ) (15, 16). By controlling the mutation diversity of GC B cells, the strength of the delivered Tfh help is a critical factor for the excessive somatic hypermutation required for the development of broadly neutralizing antibodies (17). More recently, circulating counterparts of GC Tfh cells have been identified (cTfh), a cellular compartment composed of phenotypically and functionally distinct subsets (18). Although the lineage association between the GC- and cTfh cells is not clear, analysis of cTfh dynamics has been successfully used as a surrogate of GC reactivity under certain circumstances (19).

The development of Tfh cell is a multi-step process where a combination of biological factors including antigen recognition, local inflammatory signals, co-stimulatory, and co-inhibitory signals as well as soluble mediators like cytokines and chemokines play a critical role (20). Upon antigen recognition, dendritic cells and cytokines (e.g., IL6 and IL2) are important factors for the initiation of Tfh cell differentiation (20, 21). The continuous differentiation of Tfh cells is dependent on T-B cell interactions (22, 23) and is mediated by molecules like CD80 and CD86, the ligands for CD28 (24) and ICOSL (23). This dynamic, multi-phase process, presumably leads to Tfh subsets with different functions (25, 26). Furthermore, the identification of follicular CD4 T cells with regulatory function (Tfr) (27, 28) adds to the complexity of the follicular CD4 T-cell pool. Therefore, the *in situ* heterogeneity of the Tfh cellular compartment represents a critical aspect of their biology and role in relevant diseases. Whether the phenotypic and functional heterogeneity of Tfh cells is associated with a unique compartmentalization and spatial positioning and whether these Tfh cell dynamics are stochastic or not, is not well elucidated and needs further investigation. In addition to classical Tfh markers, the use of mouse models has revealed the important role of transcriptional factors like BCL6 (29) and TOX2 (30) for Tfh cell development. Although initially considered as highly/terminally differentiated, there is growing evidence that Tfh cells display substantial flexibility and plasticity and may share characteristics of other effector CD4+ Th cell populations (31, 32). Tfh cells may also co-express other transcription factors, such as TBET, GATA3 and ROR-γt, which are expressed by Th1, Th2, and Th17 cells (33). It has been recently reported that under conditions of infection, Tfh cells express the Th1 transcription factor TBET (34). KLF2 can suppress the differentiation of Tfh cells by inducing TBET and GATA3 (35) while the absence of STAT3 accompanied by the induction of GATA3, inhibits the initial differentiation of Tfh cells (36). On the other hand, increased production of IL-4 has been associated with a GATA+BCL6+ Tfh phenotype which is regulated by Ets1 (37). Whether the expression of such transcriptional factors reflects a possible lineage association between Tfh cells and other Th effector subsets is not well understood. Furthermore, the possible role of Tfh cells expressing these transcription factors in human immune responses and disease pathogenesis needs investigation. Neoplasms derived from Tfh cells have been described. The prototype of these is angioimmunoblastic T-cell lymphoma (AITL), an overall rare lymphoma entity but one of the most common forms of neoplasms derived from mature T cells occurring in adults (38–40). AITL usually manifests as a systemic disease in adults or elderly individuals who present with generalized peripheral lymphadenopathy, often with extranodal involvement, various systemic symptoms and immune abnormalities like polyclonal hypergammaglobulinemia and Coombs-positive hemolytic anemia. It usually runs an aggressive clinical course and the median survival is <3 years (41). Tissues involved by AITL are typically enlarged by a polymorphous infiltrate comprising an abundant reactive microenvironment, and a minor component of neoplastic Tfh cells (42). Tfh lymphomas feature a characteristic mutational landscape associating mutations in epigenetic modifyers (*TET2, DNMT3A, IDH2*), *RHOA* and other T-cell receptor signaling genes (43–46).Here, we sought to characterize subsets of reactive Tfh cells in the tonsil and neoplastic Tfh cells in the context of angioimmunoblastic T cell lymphoma (AITL). A gradually increasing use of mulpiplex imaging assays both in discovery research and diagnostic pathology has been observed the recent years (47–49). Compared to traditional immunohistochemistry assays, multiplexed imaging has the advantage of the simultaneous detection of several biomarkers for the characterization of tissue elements including effector cells, stromal cells, cytokine milieu, and tissue architecture at a single focal plane. Therefore, multiplexed imaging reduces the possibility for interpretive errors, especially when rare cell populations are under investigation, while preserves valuable tissue material for further analysis. In combination with advanced computational tools, multiplexed imaging can provide a high dimension, high resolution quantitative analysis of tissue cell dynamics and microenvironment (50). We have developed a whole-slide multiplexed immunohistochemistry (mIHC) platform, employing eight antibody panels, to study and characterize the Tfh cells *in situ*. We provide data showing significant differences between tonsillar and AITL Tfh cells regarding the expression of particular markers.

## Patients and Methods

### Tissue Samples

Human tonsils were obtained from children who underwent routine tonsillectomy at the Hôpital de l’Enfance of Lausanne. The neoplastic tissue samples were retrieved from the archives of the Institute of Pathology of Lausanne University Hospital, from patients diagnosed with AITL. This monocentric research project was approved by the Ethical Committee of the Canton de Vaud, Switzerland - protocol number 123-13 and conducted according to the principles of the Declaration of Helsinki. Written informed consent was obtained from all participants.

### Staining Procedures

Sequential 4 μm thick sections from formalin-fixed, paraffin embedded (FFPE) blocks were cut and prepared on Superfrost glass slides (Thermo Scientific, Waltham, MA, Ref. J1800AMNZ), dried overnight at 37°C and stored at 4°C for conventional IHC and fluorescent mIHC staining. The slides were then heated on a metal hotplate (Stretching Table, Medite, Burgdorf, OTS 40.2025, Ref. 9064740715) at 55°C for 15 min, a melting step of paraffin for proper adherence and deparaffinization of tissue sections. All the chromogenic and fluorescent IHC were performed on the Ventana Discovery Ultra Autostainer from Roche Diagnostics.

#### Chromogenic Staining

It was performed on tissue sections using the ChromoMap DAB detection Kit (Inhibitor CM, DAB CM, H2O2 CM, Copper CM) (Ref. 750-159, Ventana Medical System, Tucson, AZ). Briefly, the slides after the melting step of paraffin, were followed by a pre-treatment heat-induced epitope retrieval at 95°C. Then, they were incubated with an inhibitor solution for 4 min to quench endogenous peroxidases activity and the Ventana dilution buffer (Ref. ADB250, from Roche) was used as a blocker for unspecific binding, for 20 min incubation at RT. Next, the slides were incubated with primary antibodies, applied either manually or with ready to use Roche Ventana dispensers at 37°C, for the validated optimum time. This was followed by incubation with horseradish peroxidase (HRP)-conjugated mouse or rabbit secondary antibodies, DAB revelation and hematoxylin counterstaining (Roche, Ref. 790-220B) for 8 min. Then, the slides were washed and dehydrated through immersion in successive baths (2 min each) with an ascending series of ethanol, followed by increasing concentration of xylene before coverslip mounting. The slides were finally scanned by the whole-slide scanner NanoZoomerS60, Hamamatsu (serial # C13210-01, Hamamatsu, Hamamatsu City, Japan).

#### Immunofluorescence Staining

The procedure consisted of consecutive rounds of antigen retrieval, antibody blocking steps (using the Opal blocking/antibody diluent solution) for non-specific binding of antibodies, staining with primary antibodies (all antibodies, clones and dilutions used in this study are listed in **Table 1**), incubation with secondary HRP-labeled antibodies, then detection with optimized fluorescent Opal tyramide signal amplification (TSA) dyes (Opal 7-color Automation IHC kit, from Akoya, Ref. NEL821001KT) and repeated antibody denaturation cycles, as previously described (51–53). Tissue sections were then counterstained with Spectral DAPI from Akoya for 4 min, rinsed in water with soap and mounted using DAKO mounting medium (Dako/Agilent, Santa Clara, CA, USA, Ref. S302380-2).

**Table 1:** Antibodies used in the multiplexed panels.

Antibody	Clone	Tissue Specificity	Species	Source	Dilution
BCL6	GI191E/A8	Nuclear	Mouse	Roche/dispenser	Prediluted
CD3	2GV6	Cytoplasmic/Membranous	Rabbit	Roche/dispenser	Prediluted
CD4	SP35	Membranous	Rabbit	Roche/dispenser	Prediluted
CD8	C8/144B	Membranous	Mouse	DAKO/M7103	1:30
CD10	SP67	Membranous	Rabbit	Roche/dispenser	Prediluted
CD21	EP 3093	Membranous	Rabbit	Roche/dispenser	Prediluted
CD30	Ber-H2	Membranous	Mouse	DAKO/M0751	1:30
CD68	PG-M1	Cytoplasmic	Mouse	DAKO/M0876	1:200
CXCL13	Polyclonal	Extracellular/Cytoplasmic	Rabbit	Thermofisher/PA5-79106	1:300
CXCR5	Polyclonal	Cytoplasmic/Membranous	Rabbit	Abcam, 46218	1:500
GATA3	L50-823	Nuclear	Mouse	Biocare Medical /405	1:25
ICOS	SP98	Membranous	Rabbit	Abcam 105227	1:50
PAX5	SP34	Nuclear	Rabbit	Roche/dispenser	Prediluted
PD-1	NAT 105	Cytoplasmic/Membranous	Mouse	Biocare Medical/3137	1:100
TBET	MRQ-46	Nuclear	Rabbit	Roche/dispenser	Prediluted

### Multispectral Scanning and Image Acquisition

Multispectral images (MSI) were acquired using the latest Vectra Polaris imaging system from Akoya. All images were recorded using a 20x magnification. The Phenochart 1.0.12 software (Akoya), a whole-slide contextual viewer with annotation capability was used for navigation around slides and identification of Regions Of Interest (ROIs), for high-resolution multispectral acquisition. A spectral library containing the emitting spectral profile of all six reporter fluorophores (in monoplex staining) and of DAPI, plus the autofluorescence signal present in FFPE tissues, obtained from a target tissue of relevant slide, was created with the Nuance Image Analysis software (Akoya). The subsequent imaging and analysis were performed on selected annotations and specific MSI fields, representing the 30–40% of the tissue. The whole tissue slides were pre-scanned at a 10x magnification and approximately 20–40 regions were selected for the acquisition of high-power (20x) MSI.

### Image Analysis and Quantification

**Figure 1:**
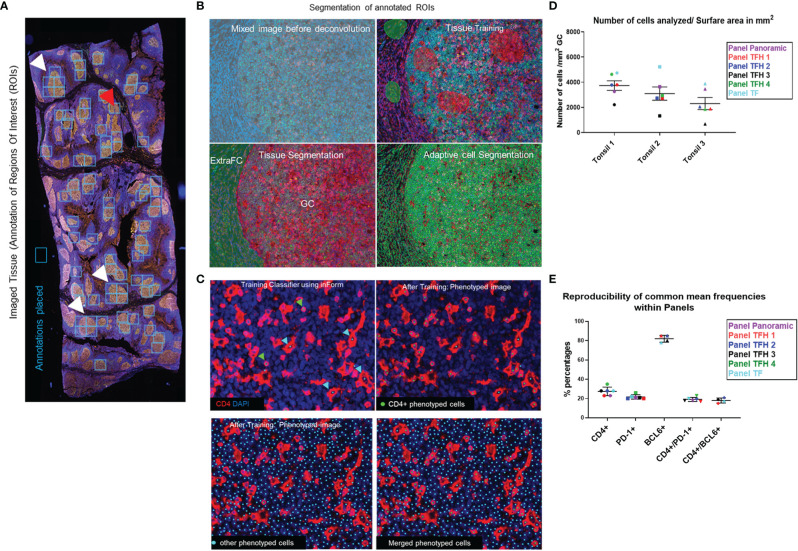
The imaging platform generates highly reproducible imaging data. **(A)** The annotated ROIs for an imaged tonsil section are shown using annotation boxes of a fixed size. White arrows mark ROIs where more than one box was applied to cover the ROI and the red arrow marks a box that covers two ROIs. **(B)** The images of annotated ROIs were unmixed and segmented into specific tissue categories (Germinal center-GC and ExtraFollicular area-ExtraFC), followed by cell segmentation based on the DAPI staining. **(C)** CD4+ T cells were identified using the phenotype approach, assigning approximately 100 cells per phenotype (green dots). Histiocytes which also express CD4, were recognized by their morphology and phenotyped as “other cells” (cyan dots) and excluded from the analysis. With this strategy, overestimation of CD4+ T cells was avoided. **(D)** The number of total cells within the total segmented germinal centers, normalized by the imaged surface area in mm2, for each of the main six multiplexed panels used are shown. **(E)** The reproducibility of calculated frequencies for representative cell phenotypes across the different multiplexed panels for one imaged tonsil is shown. Horizontal lines depict mean values of each marker calculated across the six panels and bars depict standard deviation.

MSI were analyzed using the inForm image analysis software, version 2.4.8 from Akoya. Firstly, the images were unmixed and were segmented into specific tissue ROIs [e.g., Germinal center (GC), LZ, DZ, Extrafollicular area (ExtraFC), and No Tissue] for the tonsils’ analysis, using the appropriate training components like BCL6, CD10, and PD-1 for defining the GC (**Figure 1A**). Similarly, for the Tumor and No Tissue of the lymphoma staining analysis, this tissue discrimination reflected accordingly the selected lymphoma areas from the non-neoplastic necrotic tissue or the holes lacking structural integrity. The segmentation was based on selected training markers and DAPI expression and taking into consideration the autofluorescence signal of the tissue, after manually drawing training regions on each image. The training of the segmentation algorithm was done in two ways: i) for images/panels where the “quality” of the signal was comparable among the tissues, a training pool of images was created by combining ROIs (20–40 on average) from all three tonsils and the trained algorithm was applied to segment different ROIs from the original three images and ii) for images with no comparable staining quality, ROIs from a certain tissue were used to train the algorithm and analysis of remaining ROIs from the same image. Parameters like the quality of used tissues per se and distinct features of used areas for training could introduce some bias using this path for training. In preliminary studies, it was observed that the possibility of creating false positive or negative data (segmented ROIs) by applying the first path of training across images with non-comparable staining quality was higher compared to the use of well-defined ROIs from the same tissue. ROI segmentation with training accuracy (provided by inForm) higher than 90 was used. Cell segmentation accuracy was verified by comparing manually counted imaged cells to the ones generated by the program after cell segmentation. Individual cells were segmented using the counterstained-based adaptive cell segmentation algorithm, with the help of cytoplasm and membrane markers. There might be a minor potential increase/decrease in the percentages, either because big cells/clusters of cells might not be well segmented and therefore not phenotyped as positive, or because the DAPI intensity of some cells might be very low, below the decided parameters, and consequently not get detected. In general, we found a high level of agreement (> 90%) between the manually and segmented cells. Following tissue and cell segmentation, the phenotyping configuration was used, by assigning around 100 cells to the positive phenotype for each marker, while selecting additional 100 cells characterized as “other” for the negative phenotype, choosing across several images. A similar approach using a binary classification scheme, into a positive and a negative categories was recently published (52). CD4 apart from staining CD4 T lymphocytes, also stained histiocytes. The histiocytes recognized by their size and morphology were phenotyped in CD4 immunostaining as “other cells” and therefore were excluded from the analysis (**Figure 1B**). However, because of the proximity of cells and the fact that adjacent cells might share signal, a small phenotyping error of 1% by the software in a few images was calculated. Individual algorithms were created for each marker on a set of training images, which were applied afterwards on all selected annotations of the whole-slide MSI. The quantification was based on PhenoptrReports from Akoya, an automated R-script platform, where separated merged cell segmentation data, retaining the same tissue segmentation, were created for each phenotyped marker and were consolidated afterwards. The different combinations of phenotyped populations were defined and the analysis was run creating reports, which contained the number of analyzed fields (slide summary), cell counts, cell percentages, and cell densities.

### Cell Phenotyping With Mass Cytometry (Cytof)

Cryopreserved tonsil mononuclear cells (TNMCs) were thawed and resuspended in complete RPMI medium [Gibco; Life Technologies; 10% heat-inactivated FBS (Institut de Biotechnologies Jacques Boy), 100 IU/ml penicillin, and 100 µg/ml streptomycin (BioConcept)]. Cells were washed in PBS and then incubated for 30 min with a 50 μl antibody cocktail of cell surface metal conjugated antibodies (**Table 2**). Cells were washed and fixed for 10 min at RT with 2.4% PFA. Next, cells were permeabilized for 45 min at 4°C with Foxp3 Fixation/Permeabilization kit (eBioscience), washed and stained at 4°C for 30 min with a 50 μl cocktail of T-bet, BCL-6, and GATA-3 metal conjugated antibodies. Cells were washed and fixed for 10 min at RT with 2.4% PFA. Total cells were identified by DNA intercalation (1 μM Cell-ID Intercalator, Fluidigm/DVS Science) in 2% PFA at 4°C for 1 h. The list of metal isotopes antibodies used are listed in **Table 1**. Labeled samples were assessed by the CyTOF1 instrument that was upgraded to CyTOF2 (Fluidigm) using a flow rate of 0.045 ml/min. FCS files were normalized to the EQ Four Element Calibration Beads using the CyTOF software. Data were analyzed using Fluidigm Cytobank software package (Cytobank, Mountain View, CA).

**Table 2:** Antibodies used for the Mass Cytometry phenotypic characterization of tonsillar derived cells.

Antibody	Metal	Clone	Company	Cat N.
CD8	113ln	RPA-T8	Biolegend	301018
CCR6	141Pr	11A9	DVS	3141014A
CD19	142-Nd	HIB19	DVS	3142001B
GATA-3	146Nd	TWAJ	ThermoFisher	14-9966-82
CXCR5	153-Eu	RF8B2	DVS	3153020B
CXCR3	154Sm	GO25H7	Biolegend	353718
CD45RO	165HO	UCHL1	DVS	3165011B
CD45RA	169-Tm	HI100	DVS	3169008B
CD3	170-Er	UCHT1	DVS	3170001B
PD-1	174Yb	EH12.2H7	DVS	3174020B
CD4	176-Yb	RPA-T4	DVS	3176010B
Cisplatin	198Pt	Cell-ID	DVS	201198

### Statistical Analysis

Data were analyzed using one-way ANOVA and non-parametric tests. Graphs were generated using the GraphPad Prism 8.3.0 program. The bar graphs shown in **Figures 5A, B** were generated using Python and Python modules Pandas and Seaborn. The color intensity of each bar was created based on the frequency (%) of the corresponding marker analyzed.

## Results

### Development of a Multiplex Imaging Platform for the *In Situ* Characterization of Human Tfh Cells

A comprehensive understanding of Tfh cell heterogeneity requires the simultaneous detection and analysis of several relevant molecular markers. To this end, we have used antibodies against several GC and Tfh cell markers (**Table 1**) for the development of eight multiplexed imaging panels (Immune-CD3CD8CD68CD4DAPI, Panoramic-PD-1CD8PAX5CD4CD21DAPI, TFH1-CD3PD-1CXCL13BCL6CD4DAPI, TFH2-PD-1CD10BCL6CD30CD4DAPI, TFH3-PD-1CXCR5GATA3CXCL13CD4DAPI, TFH4-PD-1CXCR5GATA3CXCL13CD4DAPI, GATA3-CD8GATA3CD4DAPI, and TF-GATA3TBETBCL6PD-1CXCR5CD4DAPI) (**Table 3**). Tonsillar tissues were used for the establishment of the panels using the experimental strategy shown in the **Supplementary Figure 1**. Single tissue staining (monoplex) was used for the optimization of each antibody used. Using the optimized experimental conditions as a reference, six-plex panels were developed, with an optimized panel shown in **Supplementary Figure 2**. Using area and cell segmentation combined with a quantitative approach (**Figures 1A–C**), we analyzed the prevalence of relevant tonsillar populations. First, the number of total cells (identified by DAPI positive nuclei) within the segmented GCs, normalized by the total GC imaged surface area in mm2, was calculated for each one of the main six multiplexed panels used. Accumulated data for three tonsils analyzed are shown in **Figure 1D**. The total number of GCs and associated GC cells analyzed might be different between panels, considering that the sections were taken at different depths in the FFPE tissue blocks. Of note, our assays generated highly reproducible data regarding the frequencies of the common markers used across the six panels (**Figure 1E** and **Supplementary Figures 3A, B**).

**Table 3:** Multiplexed panels’ information.

Panel	Order	Antibodies	Opal Fluorophores	Dilution
Immune	1	CD3	Opal 520	1/400
	2	CD8	Opal 620	1/150
	3	CD68	Opal 480	1/700
	4	CD4	Opal 690	1/150
	5	DAPI	Spectral DAPI	
Panoramic	1	PD-1	Opal 620	1/100
	2	CD8	Opal 480	1/500
	3	PAX5	Opal 570	1/100
	4	CD4	Opal 690	1/100
	5	CD21	Opal 520	1/300
	6	DAPI	Spectral DAPI	
TFH 1	1	CD3	Opal 620	1/300
	2	PD-1	Opal 650	1/200
	3	CXCL13	Opal 520	1/150
	4	BCL6	Opal 570	1/500
	5	CD4	Opal 690	1/150
	6	DAPI	Spectral DAPI	
TFH 2	1	PD-1	Opal 570	1/600
	2	CD10	Opal 650	1/300
	3	BCL6	Opal 520	1/125
	4	CD30	Opal 620	1/200
	5	CD4	Opal 690	1/150
	6	DAPI	Spectral DAPI	
TFH 3	1	PD-1	Opal 620	1/500
	2	ICOS	Opal 520	1/100
	3	BCL6	Opal 570	1/850
	4	CD10	Opal 650	1/400
	5	CD4	Opal 690	1/250
	6	DAPI	Spectral DAPI	
TFH 4	1	PD-1	Opal 620	1/350
	2	CXCR5	Opal 520	1/100
	3	GATA3	Opal 570	1/850
	4	CXCL13	Opal 650	1/400
	5	CD4	Opal 690	1/250
	6	DAPI	Spectral DAPI	
GATA3	1	CD8	Opal 620	1/400
	2	GATA3	Opal 570	1/150
	3	CD4	Opal 690	1/100
	4	DAPI	Spectral DAPI	
TF	1	GATA3	Opal 620	1:50
	2	TBET	Opal 480	1/400
	3	BCL6	Opal 520	1/100
	4	PD-1	Opal 570	1/100
	5	CXCR5	Opal 690	1/100
	6	CD4	Opal 780	1/25
	7	DAPI	Spectral DAPI	

The markers (1 to 6/7) are depicted in the order they were applied for immunostainings with their paired fluorophores and respective dilutions. T follicular cell multiplexed panel (TFH) and Transcription factor (TF) panel.

### Identification of Major Tonsillar Follicular/GC Cell Types

**Figure 2:**
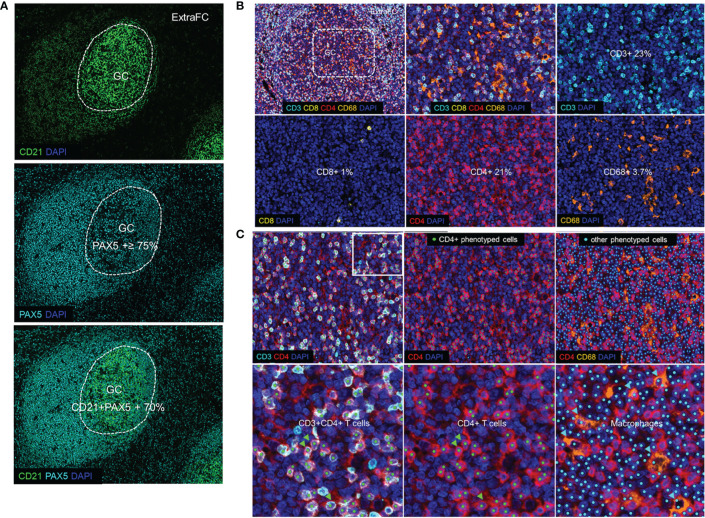
Defining germinal center and follicular immune cells. **(A)** The expression pattern of CD21 and PAX5 (Panoramic panel) guided the identification of the GC versus the ExtraFC areas. **(B)** A representative follicular area showing the localization of CD3 (cyan), CD8 (yellow), CD4 (red) and CD68 (orange) and DAPI positive events (upper left). Individual cell types in a zoomed GC area (dotted line) are shown. **(C)** Phenotyped images of a follicular area as well as a zoomed area (inset) showing CD3+CD4+ cells (CD4+ T lymphocytes) (green arrowheads) and other CD3-CD4+CD68+ cells which were considered as stained macrophages (cyan arrowheads) and excluded from the quantification of the total CD4+ T lymphocytes.

First, we analyzed the main follicular and particularly GC cell types, including B cells, follicular dendritic cells (FDC), T cells, and macrophages (tingible body macrophages). As expected, the majority of cells expressed high levels of CD21 (abundant in B cells) and PAX5 (a transcription factor found in B cells) (**Figure 2A**). Specifically, approximately 79% of total GC cells were PAX5+ and 70% of PAX5+ cells co-expressed CD21 (**Supplementary Figure 3A**). We identified the FDC backbone of the GC area based on its circumferential meshwork staining pattern for CD21 (**Figure 2A**). The expression pattern of CD21 and PAX5 guided the identification of GC and non-GC follicular areas (**Figure 2A**). Regarding T cells, CD3+ cells made up the 24% of total cells (panel TFH 1) in the GC (**Figures 2B, C** and **Supplementary Figure 3A**). We identified and quantified “real” CD4+ T lymphocytes by excluding histiocytes, being also positive to the CD4 antibody, as explained in the Methods section and shown in **Figure 2C**. Approximately, 23% of the total GC cells were positive for CD4 (panel TFH 1) (**Figure 2B** and **Supplementary Figure 3A**). Around 1% of the total GC cells were identified as CD3+CD8+ (**Figure 2B** and **Supplementary Figure 3A**). CD68 that labels macrophages appeared to highlight scattered macrophages within the GC at a percentage of 3.7% (**Figures 2B, C**). As expected, our collective analysis focusing on follicular CD4+ T cell revealed the dominance of subsets expressing classic Tfh markers (e.g., PD-1, BCL6, CXCR5, and ICOS) (**Table 4**). Altogether, our data show that the described platform provides solid data for the *in situ* analysis of GC immune cells, especially Tfh cells.

**Table 4:** Collective data from all six developed multiplex panels, with phenotypes combinations after analysis of three tonsils and three selected AITL cases, with reference to the GC CD4+PD-1+ cells in tonsils and to CD4+PD-1+ cells in the Tumor niche in AITL cases.

(%) Percentages	Tonsil 1	Tonsil 2	Tonsil 3	Averages	AITL 1	AITL 2	AITL 3	Averages
	From Total GC cells				From Total Tumor cells			
**CD4+PD-1+**	**24.66**	**16.16**	**16.66**	**19.16**	**5.83**	**24.0**	**50.16**	**26.66**
**(%) Percentages with reference to CD4+PD-1+ cell**	**in the GC**				**in the Tumor niche**			
**CD3+CD4+PD-1+**	94.20	95.0	92.50	93.90	97.20	96.40	90.80	94.80
**CD4+PD-1+BCL6+**	77.47	71.52	71.55	73.52	49.90	43.25	32.57	41.91
**CD4+PD-1+CD10+**	54.95	67.30	69.55	63.93	32.45	24.35	21.65	26.15
**CD4+PD-1+ICOS+**	78.90	80.40	60.30	73.20	71.50	88.40	71.40	77.10
**CD4+PD-1+CXCR5+**	85.20	83.15	86.40	84.92	63.80	44.60	60.25	56.22
**CD4+PD-1+CXCL13+**	23.15	25.65	26.95	25.25	56.40	28.80	65.15	50.12
**CD4+PD-1+CD30+**	0.10	0.01	0.01	0.04	8.0	35.50	64.70	36.07
**CD4+PD-1+GATA3+**	63.0	59.65	52.1	58.25	32.75	37.05	7.85	25.88
**CD4+PD-1+TBET+**	0.10	0	0	0.03	4.60	0.70	0.50	1.93
**CD4+PD-1+BCL6+CD10+**	48.35	53.60	58.45	53.46	19.10	12.85	5.0	12.32
**CD4+PD-1+BCL6+ICOS+**	62.0	54.60	46.50	54.36	37.10	41.0	22.40	33.50
**CD4+PD-1+CD10+ICOS+**	31.50	61.40	35	42.63	27.10	19.60	19.40	22.03
**CD4+PD-1+BCL6+CD30+**	0.10	0	0	0.03	4.30	26.10	31.10	20.50
**CD4+PD-1+CD10+CD30+**	0	0	0	0	1.80	11.20	3.80	5.60
**CD4+PD-1+BCL6+CXCL13+**	12.20	20	13.40	15.20	16.70	8.10	19.70	14.83
**CD4+PD-1+CXCR5+CXCL13+**	26.70	15.50	19.10	20.43	37.20	14.80	42.60	31.53
**CD4+PD-1+GATA3+CXCL13+**	16.10	11.40	11.60	13.03	9.10	8.50	3.50	7.03
**CD4+PD-1+CXCR5+GATA3+**	51.50	48.0	45.20	48.23	22.65	13.90	4.70	13.75
**CD4+PD-1+CXCR5+BCL6+**	57.80	64.30	64.80	62.30	51.70	26.50	35.30	37.83
**CD4+PD-1+CXCR5+TBET+**	0	0	0	0	2.80	0.20	0.20	1.06
**CD4+PD-1+BCL6+TBET+**	0	0	0	0	2.0	0	0	0.66
**CD4+PD-1+BCL6+GATA3+**	36.0	34.20	37.30	35.83	27.40	20.60	3.20	17.06
**CD4+PD-1+TBET+GATA3+**	0	0	0	0	1.70	0.30	0	0.66
**CD4+PD-1+BCL6+CD10+ICOS+**	26.0	43.20	27.40	32.20	16.20	10.80	5.40	10.80
**CD4+PD-1+BCL6+CD10+CD30+**	0	0	0	0	1.80	11.20	3.80	5.60
**CD3+CD4+PD-1+BCL6+CXCL13+**	11.60	19	12.40	14.33	16.70	7.90	19.40	14.66
**CD4+PD-1+CXCR5+CXCL13+GATA3+**	13.70	7.70	6.20	9.20	4.90	2.80	1.50	3.066
**CD4+PD-1+CXCR5+BCL6+TBET+**	0	0	0	0	1.50	0	0	0.50
**CD4+PD-1+CXCR5+BCL6+GATA3+**	32.10	32.20	37.20	33.83	24.10	11.0	2.90	12.66
**CD4+PD-1+CXCR5+TBET+GATA3+**	0	0	0	0	0.90	0	0	0.30
**CD4+PD-1+BCL6+TBET+GATA3+**	0	0	0	0	0.70	0	0	0.23
**CD4+PD-1+CXCR5+BCL6+TBET+GATA3+**	0	0	0	0	0.60	0	0	0.20

### Characterization of Tonsillar Tfh Cells

**Figure 3:**
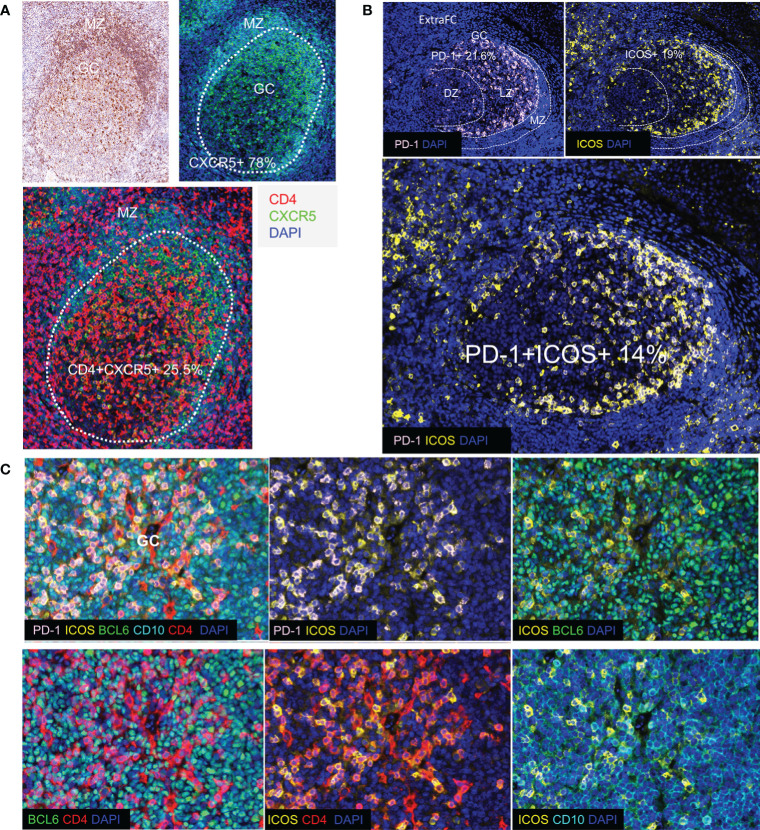
Detection of Tfh cell subsets in human tonsils. **(A)** CXCR5+ cells and their localization in human tonsil assessed by IHC (upper left panel) and by immunofluorescence (upper right and lower panels). The borders of GC are shown by dotted lines. CXCR5 was strongly expressed especially in the mantle zone (MZ). CXCR5 is expressed in B cells (CD4-CXCR5+) as well as in a subset of CD4+T cells in the GC. **(B)** Immunofluorescence image showing distinct follicular areas (MZ, DZ, LZ). The MZ area is indicated by dotted lines (left panel). Polarization of PD-1+ cells in the LZ of the GC was observed in most of the follicles analyzed. The localization of ICOS (right panel) and ICOS+PD-1+ (lower panel) cells is shown. A similar polarization pattern was observed for both antigens. **(C)** Representative images showing the co-expression of Tfh cell markers (PD-1, ICOS, BCL6, and CD10) in a GC (panel TFH 3).

Tfh cells are defined by a unique phenotype characterized by the high surface expression of several markers including CXCR5 (54), PD-1 (55), and ICOS (56). We started investigating the *in situ* heterogeneity of Tfh cell using both surface and nuclear markers. We found that 78% of total GC cells were positive for CXCR5 (**Supplementary Figure 3A**). Using three different thresholds (low, dim, and high, defined by intensity counts of 5, 7, and 9, respectively), we found that CXCR5^high^ cells were located in the GC and at the Mantle Zone (MZ) admixed with medium and low CXCR5 expressing cells, while CXCR5^low^ cells were detected mainly in the ExtraFC area (**Figure 3A** and **Supplementary Figures 4A, B, D**). Quantitative analysis revealed that approximately 20% of total GC cells expressed a PD1+CD4+ phenotype (**Table 4**) while 85% of PD1+CD4+ T cells were CXCR5+ (data from TFH 4 and TF panels, **Table 4**). Therefore, simultaneous analysis of CD4 and PD-1 is a reliable way for identification of GC Tfh cells. With regard to their localization, a distinct polarization of PD-1+ CD4+ T cells towards the apical light zone of the GC was observed (**Figure 3B**). We observed that the vast majority of CD4 T cells in the follicular and GC area express a PD-1^high^/PD-1^dim^ phenotype compared to extrafollicular CD4 T cells which are PD-1^low^ (**Supplementary Figures 4C, D**). Similar to PD-1, ICOS+ cells were polarized in the outer rim of the GC adjacent to the MZ (**Figure 3B**). Quantitative analysis of ICOS expression showed a wide overlap with PD-1 (Tfh cells co-expressed ICOS at 73.2%) (**Table 4**) in agreement with the critical role of ICOS+ Tfh cells in GC reactivity (23). Analysis of BCL6, a transcription factor critical for the development of Tfh cells (57) revealed a wide expression in the GC area. In line with its expression in B and Tfh cells, BCL6 was expressed in approximately 80% of total cells in the GC (**Figure 3C** and **Supplementary Figures 2B**, **3A**) while approximately 73% of Tfh cells were positive for BCL6 (**Table 4**). CD10, a glycoprotein that is expressed on GC B cells and a subset of Tfh cells (58) exhibited a positive diffused cytoplasmic and membranous signal inside the GC at a percentage of 82% from the total cells (**Figure 3C** and **Supplementary Figure 2B**). Furthermore, 64% of Tfh cells co-expressed CD10, while 53.5% of Tfh cells expressed a CD4+PD-1+BCL6+CD10+ phenotype (**Table 4**). A considerable expression of CXCL13, the ligand of CXCR5, was also found with 25% of Tfh cells and 15% of CD4+PD-1+BCL6+ cells staining for CXCL13 (**Table 4**). Altogether, our combinatorial analysis revealed a heterogeneous pool of Tfh cells with respect to the expression of surface (PD-1, ICOS, CXCR5, and CD10), nuclear (BCL6) and functional (CXCL13) markers.

### *In Situ* Phenotyping Reveals a Significant Expression of GATA3 in Tfh Cells

**Figure 4:**
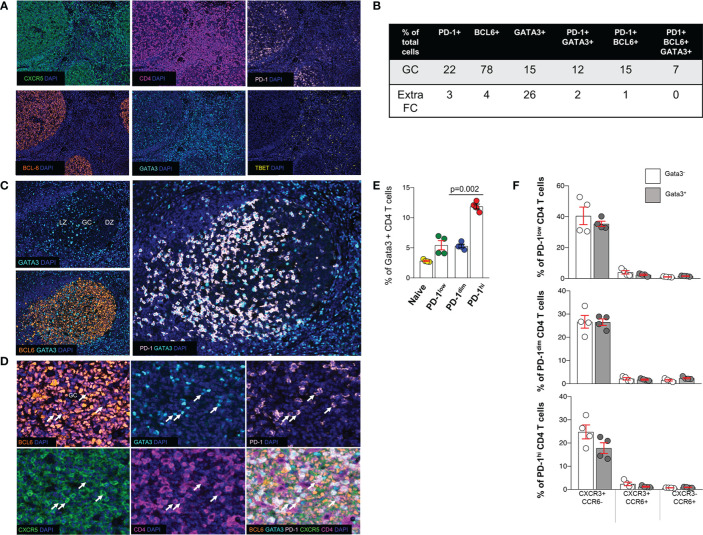
Expression of transcription factors in tonsillar Tfh cell subsets. **(A)** Representative, low-magnification immunofluorescent images showing the distribution of CXCR5, PD-1, BCL6, GATA3 and TBET in a tonsil. **(B)** The prevalence (expressed as % of total cells in the corresponding area) of cells positive for PD-1, BCL6, GATA3 or their combinations in GCs and ExtraFC areas is shown. Follicular areas (n = 5) from three tonsils were analyzed. **(C)** The distribution of GATA3+ cells in GC areas, judged by the BCL6 staining profile, is shown. A polarization of GATA3+ cells towards the LZ, mirroring the distribution of PD-1 expression was consistently was found. **(D)** Representative images showing the co-expression of relevant markers (TF panel) in tonsil GC. CD4+PD-1+CXCR5+BCL6+GATA3+ cells observed in the GC compartment are pointed by white arrows. **(E)** Bar graph showing the frequency of GATA3+ cells in different tonsillar CD4 T cell subsets, analyzed by Cytof. The pair t-test was used for the statistical analysis. **(F)** Bar graphs depicting the frequency of CATA3+(GATA3-) PD1^lo^, PD1^dim^ and PD1^hi^ CD4 T cells and with respect to the expression of CXCR3 and CCR6.

Next, we sought to investigate the expression of TBET (59) and GATA3 (60), two major CD4 T-lineage transcription factors, in the follicular area. Application of a 6-plex panel revealed a differential localization between BCL6, GATA3 and TBET (**Figure 4A**). In contrast to BCL6, a wider distribution, both in follicular and ExtraFC areas, was observed for GATA3 while TBET was almost exclusively found in the ExtraFC area (**Figures 4A, B**). Our quantitative imaging showed that CD4+PD-1+BCL6+GATA3+ T cells were exclusively localized in the GC area (**Figure 4B**). Interestingly, we observed a polarization of GATA3+ cells towards the light zone, mirroring the distribution of PD-1 expression (**Figure 4C**). Analysis of specific populations showed a heterogeneity of Tfh cells with respect to BCL6 and GATA3 expression. Specifically, 58% of Tfh cells express GATA3 while only 35.8% of them co-express BCL6 and GATA3 (**Figure 4D** and **Table 4**). Inclusion of CXCR5 in our analysis revealed even higher heterogeneity of the Tfh cell pool with 33.8% of Tfh cells been CXCR5+BCL6+GATA3+ (**Table 4**). We sought to further characterize the phenotype of GATA3+ Tfh cells using a Cytof assay (**Supplementary Figure 5**). Our data showed a significantly higher frequency of GATA3+ cells in the PD1^hi^ Tfh cells compared to other tonsillar CD4 T cell subsets (**Figure 4E**). The majority of cells expressed a CXCR3-CCR6- phenotype in all CD4 T cell subsets analyzed, followed by the CXCR3+CCR6- phenotype (**Figure 4F**). A low frequency of cells expressed a CXCR3+CCR6+ or CXCR3-CCR6+ phenotype (**Figure 4F**). Altogether, our data show that determination of Tfh cell heterogeneity requires investigation of both surface receptors and transcriptional factors, especially GATA3.

### Identification of Tonsillar Tfh Cell Tissue Signatures

**Figure 5:**
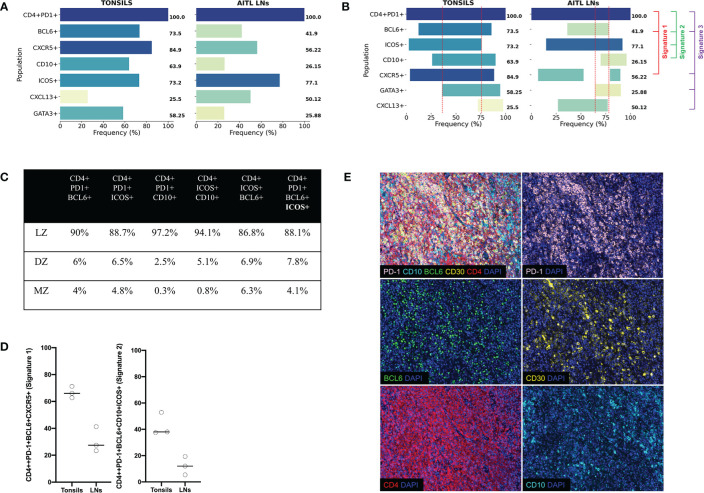
Tonsillar and AITL LN Tfh cell express different core signatures. **(A)** Bar graphs showing the expression (%) of individual Tfh cell markers with reference (expression at 100%) to CD4+PD-1+ cells from the average of three human tonsils and three AITL lymph nodes. Collective data of all six multiplexed developed panels were used. **(B)** Bar graphs showing the co-expression of individual markers defining particular Tfh cell signatures (Signature 1 CD4+PD-1+BCL6+CXCR5+ used panels: TFH1, TFH2, TFH3, TFH4, and TF, Signature 2 CD4+PD-1+BCL6+ICOS+CD10+, used panels: TFH1, TFH2, TFH3, and TF, and Signature 3 CD4+PD-1+BCL6+CXCR5+CXCL13+GATA3+, used panels: TFH1, TFH2, TFH3, TFH4, and TF, 3). The dotted red lines define the area of co-expression for the most abundant Tfh markers (PD1, BCL6, ICOS, CD10, CXCR5, and GATA3) referred to as “core signature”. **(C)** Pooled data showing the distribution of Tfh cells expressing the signature 1 in different tonsillar ROIs. **(D)** Dot plots showing the frequency of Tfh cells expressing the signatures 1 and 2. Cells from three tonsils and three lymph nodes were analyzed. **(E)** Representative immunofluorescence images of AITL 2 case stained with the TFH 2 panel. A merged image and the staining of individual markers are shown.

Combining the information collected from all developed panels can provide comprehensive phenotypic signatures of Tfh cells. To this end, CD4 and PD-1 were used as “reference” markers (expression 100%). As it was expected, CXCR5 and BCL6 and ICOS were the most widely expressed markers (**Figure 5A**, left panel). We defined two tonsillar Tfh phenotypic signatures based on the prevalence of dominant markers CD4+PD-1+BCL6+CXCR5+ (Signature 1, 69.4% of CD4+PD-1+cells), CD4+PD-1+BCL6+ICOS+CD10+ (Signature 2, 42.8% of CD4+PD1+ cells), while a less frequent signature found when the CXCL13 and GATA3 was introduced (CD4+PD-1+CXCR5+BCL6+CXCL13+GATA3+, Signature 3, 13.7% of CD4+PD-1+cells) (**Figure 5B**, left panel). Next, the frequency of Tfh cells expressing the signature 1 with respect to their localization in DZ, LZ, and MZ areas was analyzed. The vast majority of these events located in the LZ with a minority (around 4–6%) found in DZ or MZ (**Figure 5C**). Therefore, our imaging platform could provide the base for further understanding of the heterogeneity of Tfh cell in disease.

### Different Tfh Core Signatures Between Tonsillar and Neoplastic Lymph Node Tissues

Next, we aimed to assess the Tfh phenotypic signatures in lymph nodes from three AITL individuals with different mutational status (**Table 5**). As expected an altered distribution of Tfh cells was found compared to tonsils (**Supplementary Figure 6**). Specifically, a polarized Tfh cell distribution within GC as seen in tonsils was missing (**Supplementary Figure 6**) while a less structured clustering of Tfh cells was evident in areas characterized by sequestration of BCL6 positive cells (**Figure 5D**), a profile associated with the frequency of tumor cells inferred from the VAF of tumor specific mutations (RHOA G17V VAF and IDH2 VAF) (**Table 4** and **Supplementary Figure 6**). Furthermore, the skewed distribution of BCL6, GATA3, and TBET found in tonsils was missing in AITL LNs (**Supplementary Figure 7A**). Similar to tonsils, application of the mIHC panels generated highly reproducible data for the AITL LNs (**Supplementary Figure 7B**). As it was expected, a higher frequency of PD1+CD4+ T cells was found in LNs (29%) compared to tonsil GC (19%) (**Table 4** and **Supplementary Figure 8A**). A heterogeneity across the three cases was observed, concerning especially the CXCL13 and GATA3 expression (**Supplementary Figure 8A**). CD4 T cells was the dominant immune cell type in the tumor area while other immune cells (CD8, CD21) were less frequent (**Supplementary Figure 8A**). Although the sample size analyzed was not sufficient for the extraction of statistically significant data, we consistently found differences between tonsils and lymph nodes when several Tfh biomarkers were analyzed (**Figure 5A** and **Table 3**). Specifically, we found lower frequency of Tfh cells expressing Bcl6 (41.9 vs 73.5), CD10 (26.15 vs 63.9), and GATA3 (25.88 vs 58.25) in LNs compared to tonsils (**Figure 5A** and **Supplementary Figure 8B**). In contrast, a higher frequency of CXCL13+ Tfh cells was found in LNs compared to tonsils (50.12 vs 25.5) (**Figure 5A** and **Supplementary Figure 8B**). Approximately 5% of non-T cells in the tumor area were CD30+. Interestingly, a relatively high frequency of Tfh cells were positive for CD30, a receptor often found in AILT (61–63), in LNs (36%) while normal Tfh cells in tonsil GC very rarely coexpressed CD30 (0.04%) (**Table 4**). Analysis of the three aforementioned Tfh cell signatures revealed a different pattern in AITL compared to tonsils (**Figure 5B**). Interestingly, a higher frequency of Tfh cells was found to express signature 1 and 2 (**Figures 5B, D**) while the difference was less evident for the signature 3 (13.7 vs 1.5) in tonsils compared to AITL LNs (**Figure 5B**). Therefore, the altered, less structured localization associated with a differential expression of Tfh cell subsets could represent biological factors contributing to the heterogeneity of the Tfh cell compartment in AITL patients.

**Table 5:** Demographic data, tissue information as well as the mutational status of the three AITL LNs used are shown.

Case		AITL 1	AITL 2	AITL 3
Gendre/Age		M/70	F/83	M/77
Lymph Node		cervical	inguinal	Not Specified
RHOA	G17V VAF	3%	7%	6.9%
TET2	(exon3) VAF	3%	6%	11.4%
TET2	(exon10) VAF	4%	11%	15.4%
DNMT3A	(exon15) VAF	N/A	11%	N/A
IDH2	(exon4) VAF	N/A	9%	7.8%

VAF, Variant Allele Frequency.

## Discussion

Similar to a previous study (64), we have employed a multiplexed immunofluorescence approach to assess the *in situ* distribution of Tfh cells in human lymphoid tissues. We have built standardized protocols comprising eight panels including several surface and nuclear markers to quantitatively evaluate the frequencies, spatial distribution, co-localization, and positioning of Tfh cells in healthy and neoplastic tissues. Despite their unique anatomical localization compared to LNs, tonsils contain numerous follicular structures with relatively high frequencies of Tfh cells which have been extensively investigated (65–69). In line with previous studies (65, 66), we found a considerable amount of CD3 T cells in the GC area with the vast majority of them being CD4+ T cells. High expression of PD-1, a widely used marker for Tfh cell identification, was exclusively found in GC CD4 T cells with approximately 75% of them characterized by a PD-1+ phenotype. No PD-1 expression was found in GC PAX5+ and FDC cells. Our data are in agreement with previous studies (67, 69) and confirm the value of PD-1 and CD4 as reference markers for the *in situ* identification of Tfh cells. The orchestrated downregulation of CCR7 and upregulation of CXCR5 is a driving force for the trafficking of CD4 T cells into the follicular and GC areas (70). Published data regarding the expression of CXCR5 in lymphoid tissues and blood have been essentially derived from flow cytometry analyses and frozen section immunohistochemistry (71, 72), which have consistently shown high levels of expression in Tfh cells particularly the GC ones. Our work is the first to provide documentation of CXCR5 expression in Tfh cells by using IHC/IF assays on FFPE tissues allowing for a good morphologic discrimination and preservation of the tissue structure. We found that CXCR5 was expressed by both B cells, in accordance with flow cytometry data (73), as well as by a subset of CD4+ T cells in lymphoid tissues, and from our collective data the GC CD4+PD-1+ cells co expressed CXCR5 at 85% in alignment with the literature (2). ICOS, a critical factor for Tfh cell differentiation/maintenance (56, 74) is highly expressed in tonsillar Tfh cells, and accordingly we found high expression of ICOS in the majority of GC PD-1+CD4+ T cells (75, 76). Interestingly, a wide overlap between PD-1 and ICOS expression in Tfh cells was observed pointing to a coordinated function of these receptors. Investigation of their ligands (PD-L1, ICOSL) expression could provide cues for the mode and time order of their function. The simultaneous expression of PD-1 and ICOS can further inform for the local compartmentalization of Tfh cells. By detecting different levels of single intensity of PD-1, along with CXCR5, we noticed a tendency of Tfh cells with PD-1 and CXCR5 high expression to be located at the outer rim of the GC, near to the MZ. Therefore, our imaging platform can provide meaningful data for the frequency/numbers of Tfh cell populations with respect to their local compartmentalization.

The combined analysis of several markers revealed the presence of tonsillar Tfh cell subsets at least at the phenotypic level. We identified a “core” of markers (PD-1, ICOS, CXCR5, CD10, BCL6, and GATA3, **Figure 5A**) that we call the “core signature” and could be used in future studies for the tracking of Tfh cell subsets at tissue level. Whether this localization profile is associated with different functionality of the described Tfh cell subsets is not known and needs further investigation. Our imaging analysis revealed that approximately 20% of Tfh cells were positive for CXCL-13. CXCL-13, the ligand of CXCR5, is produced by several cell types both in the extrafollicular (77) and intrafollicular area (78). Its autocrine and paracrine functions make it a major player for the organization of B-cell follicles and GCs. Although our assays cannot address whether the CXCL13 is produced or just bound to Tfh cells, use of functional markers like the CXCL13 chemokine in combination with the aforementioned receptors can guide the investigation of Tfh heterogeneity both at phenotypic and functional level.

The lineage origin and differentiation commitment of human Tfh cells is poorly known. Studies using mouse models have shown that naïve CD4 T cells acquire lineage-depend effector functions after encounter of a foreign antigen presented by antigen presenting cells (APC). The initial description of Th1, Th2, and Th17 (79) has been followed by the identification of several other CD4+ T cell types including Treg, Th9, and Tfh subtypes (20, 80). It is generally considered that the commitment into the different subtypes is dictated by the expression of lineage-specifying transcription factors such as TBET, GATA3, BCL6, or RORγt conferring to the subtype unique sets of phenotypes, functions, and selective effector cytokines production while suppressing their differentiation into an alternative subtype (81–85). GATA3 is a transcription factor important for the differentiation of several cell lineages including breast epithelia and urothelial cells (86) as well as subsets of T lymphocytes for Th2 maturation (85). Its role in Tfh cell differentiation is not well understood as both positive (87, 88) and negative roles (35) have been proposed. Our data show that about one third of CD4+ PD1+ GC cells in the tonsil co expressed CXCR5, BCL6 and GATA3 implying an important role for GATA3 for human Tfh cell development. Our Cytof analysis revealed that the majority of tonsillar GATA3+ Tfh cells express a Tfh2 phenotype (CXCR3-CCR6-), while an evident population (approximately 20%) express a Tfh1 (CXCR3+CCR6-) profile (18). Whether the heterogeneity of circulating Tfh cells (89) mirrors the one found in follicular Tfh cells is not known. The lineage relationship between Tfh subsets expressing similar profiles (Tfh1, Tfh2) in blood and lymphoid organs remains to be elucidated.

Our data are in line with recent studies suggesting that the orchestrated activity of multiple transcription factors rather than a “unique, master” regulator is needed for the development of T helper cell subtypes (90, 91). A recently proposed model implies that an epigenetic mechanism orchestrating a transient expression of TBET, GATA3, or RORγt together with BCL6 at early stage of differentiation in Tfh cells, could justify their underlying ability to produce other lineages’ specific cytokines (92). Our data showed a trend for GATA3 polarization towards the light zone. The expression of GATA3 in human Tfh cell subsets raising questions regarding their origin from further Th2 differentiated cells or the irreversible differentiation fate of the CD4 T cells. In fact, increasing evidence supports an emerging model suggesting that upon changing conditions of the microenvironment in their surroundings, effector CD4 T cells could effectively rewire their lineage-specific functional program having previously preserved traces of other lineage-defining factors (31, 92).

Our previous work has provided critical data showing the origin of AITL from Tfh cells (42). Others later expanded these observations, resulting in the concept now adopted by the WHO classification, that the nodal lymphomas of Tfh derivation comprise a spectrum of diseases including AITL, follicular T-cell lymphoma, and other nodal lymphomas with a Tfh phenotype. Importantly, since we have described the mutational status of AITL as well as other lymphomas of Tfh origin (93), we were thus interested to illustrate the frequencies and co-expressions of Tfh neoplastic cells *in situ*. The calculated frequencies of Tfh cells in AITL LNs (**Table 3**) were higher than the estimated neoplastic cells (**Table 4**). Despite the small size of the analyzed LNs, an association between the prevalence of these two cell types was observed. Further studies aiming to establish such correlation are needed. Although the frequency of Tfh cells was higher in AITL compared to tonsils, the percentage of AITL Tfh cells expressing a BCL6^hi^ phenotype was reduced. Whether this is due to an over expansion of the AITL BCL6^lo^ Tfh cells or a defected expression of BCL6 per se needs further investigation. Furthermore, the lower frequency of CXCR5^hi^ AITL Tfh cells was associated with a higher frequency of CXCL13^hi^ Tfh cells compared to tonsils. We hypothesize that this profile could represent a higher rate of recognition of CXCR5 by its ligand ultimately leading to downregulation/recycling of the receptor. In line with this, a relatively high frequency of Tfh cells co-expressing CXCR5 and CXCL13 was found in AITL compared to tonsils, suggesting an important role for the CXCR5/CXCl13 axis as regulator of the Tfh cell trafficking and positioning in AITL LNs. CD30, a receptor found on activated T cells and widely used for diagnosis of peripheral T lymphomas (61, 62, 94), was readily found in AITL Tfh cells but it was almost absent in their tonsillar counterparts. Interestingly, the majority of CD30^hi^ Tfh cells were expressing a BCL6^hi^ phenotype too, indicating a possible role of CD30 in the differentiation process of AITL Tfh cells. Although the WHO criteria specifically state that expression of at least three Tfh markers is required for assignment of a Tfh phenotype (38, 95), we detected a high level of heterogeneity concerning six Tfh markers, which were robustly co expressed in the tumor niche, that is to say CD4, PD-1, CXCR5, ICOS, Bcl6, and CXCL13. In a recent study, two AITL cases were analyzed using multiplexed quantitative immunohistochemistry techniques and one panel to simultaneously stain for a collection of relevant markers in lymphoid tissue to determine the expression of marker of interest in malignant cells, such as the CD4, PD-1, and BCL6. On average, 45.5% of the CD4+ T cells in the samples were positive for PD-1, while 26% were positive for BCL6 (96). Despite the limited number of samples analyzed, which precludes the extraction of robust conclusions, we found a differential representation of particular Tfh cell subtypes/signatures between tonsils and AILT LNs, a finding that could trigger further investigation of the Tfh heterogeneity in relevant subjects. The differential representation of Tfh cell subsets was also associated with an altered distribution of Tfh cells within the tumor area compared to tonsils too. An atypical, less structured distribution of Tfh cells in AILT was more evident in the donors with higher frequency of tumor cells and higher prevalence of unique mutations found in AILT donors. Our data urge further combined analysis of particular Tfh cell populations, their spatial positioning and genetic background of neoplastic cells that could establish an association between these biological parameters and a possible role in disease progression.

In conclusion, using tonsils, we have optimized a multiplex IF protocol for mapping the cellular distribution by multiple staining in a single section, conserving limited clinical material, while enabling spatial resolution of co-localized antigens. Despite the limited number of tissues analyzed, our data provide a proof of concept for such tissue analysis aiming to understand the heterogeneity of the Tfh cell compartment in health and disease. The described phenotypes and tissue signatures can provide the base for the development of antibody panels with higher complexity and integration with other imaging platforms (e.g., multiplexed confocal imaging) for further investigation of Tfh cell heterogeneity. Our data suggest that the combined investigation of spatial positioning and *in situ* phenotyping (surface receptors, functional biomarkers, nuclear factors) of Tfh cells can provide a comprehensive analysis for their heterogeneity and plasticity in relevant diseases like AILT. This is particularly crucial for diagnostics and classifying disease state in the future.

## Data Availability Statement

The original contributions presented in the study are included in the article/**Supplementary Material**. Further inquiries can be directed to the corresponding author.

## Ethics Statement

The studies involving human participants were reviewed and approved by Ethical Committee of the Canton de Vaud, Switzerland - protocol number 123-13. The patients/participants provided their written informed consent to participate in this study.

## Author Contributions

LL conceived the study. KI, D-RN, AN, CF, and SF performed experiments and analyzed data. GP provided intellectual input and edited the manuscript. KI, CP, and LL wrote the manuscript. All authors contributed to the article and approved the submitted version.

## Funding

This study was funded by the SNF grant (310030_172954).

## Conflict of Interest

The authors declare that the research was conducted in the absence of any commercial or financial relationships that could be construed as a potential conflict of interest.

## Supplementary Material

Supplementary Figure 1 Overview of the workflow of advanced multiplexed immunohistochemistry from staining to image analysis.

Supplementary Figure 2 Monoplexed and multiplexed immunohistochemistry in tonsils. **(A)** Representative examples of histological evaluation from IHC (upper panel) and monoplex IF (lower panel) with optimal staining pattern, intensity, and morphology. **(B)** Representative images generated with the TFH 3 mIHC panel.

Supplementary Figure 3 **(A)** Mean expression of individual markers in the three tonsils (expressed as % of the GC cells) from the different mIHC panels. **(B)** Expression of markers used in different tonsils among all mIHC panels. Statistical analyses were performed using one-way ANOVA and non-parametric tests. ****P* ≤ 0.001, ***P* ≤ 0.01, **P* ≤ 0.05.

Supplementary Figure 4 **(A)** Identification of extrafollicular (ExtraFC), germinal center (GC) and mantle zone (MZ) areas in a tonsillar follicle (right panel). A merged image with all markers used is shown in the left panel. Follicular Tfh cells divided into two subsets based on their localization, GC, and follicular mantle zone cells. **(B)** Cells with differential expression of CXCR5 (high-red, medium-orange, and low-yellow) in the three different compartments are shown. **(C)** The localization of PD-1 high (red), medium (orange), and low (yellow) cells in the same follicular areas is shown. **(D)** quantification of follicular cells with respect to expression level of PD-1 and CXCR5 in GC, MZ and ExtraFC is shown.

Supplementary Figure 5 The gating scheme for the Cytof analysis of tonsillar Tfh cell subsets is shown. N tonsils were used for the analysis.

Supplementary Figure 6 An overview of the BCL6 staining for the whole LN imaged area from each donor is shown (upper row). Our analysis program (inFORM) allows the overview presentation only for imaging data before processing (unmixing). BCL6 label was the only marker used without crosstalk signal with the rest of the markers. The expression of BCL6 (in red) and PD1 (green) (post-processing data) in a small area (red boxes) as well as zoomed images (white boxes) are shown for each donor. The images show that the sequestration of BCL6 is associated with that of TFH (PD1+) cells.

Supplementary Figure 7 **(A)** Representative immunofluorescent images showing the localization of PD1, BCL6, GATA3, and TBET in the tumor area of an AITL LN. **(B)** Reproducibility of common mean frequencies with standard deviation in lymphoma cases among the different panels.

Supplementary Figure 8 **(A)** Mean expression (%) of immune and lymphocyte markers for the three AITL lymphoma cases analyzed in all developed panels of the study with reference to total tumor cells. **(B)** Dot plots showing the frequency of Tfh cells expressing a BCL6, CD10, GATA3, or CXCL13 positive phenotype. Cells from three tonsils and lymph nodes were analyzed.
