# Supplementary material for: In Situ Characterization of Follicular Helper CD4 T Cells Using Multiplexed Imaging
Source: Front Immunol. 2021 Feb 3;11:607626. doi: 10.3389/fimmu.2020.607626 (PMC7901994; doi:10.3389/fimmu.2020.607626)
Supplement: Supplementary Figure 1 — Overview of the workflow of advanced multiplexed immunohistochemistry from staining to image analysis. [file Presentation_1.pptx]

## Slide 1
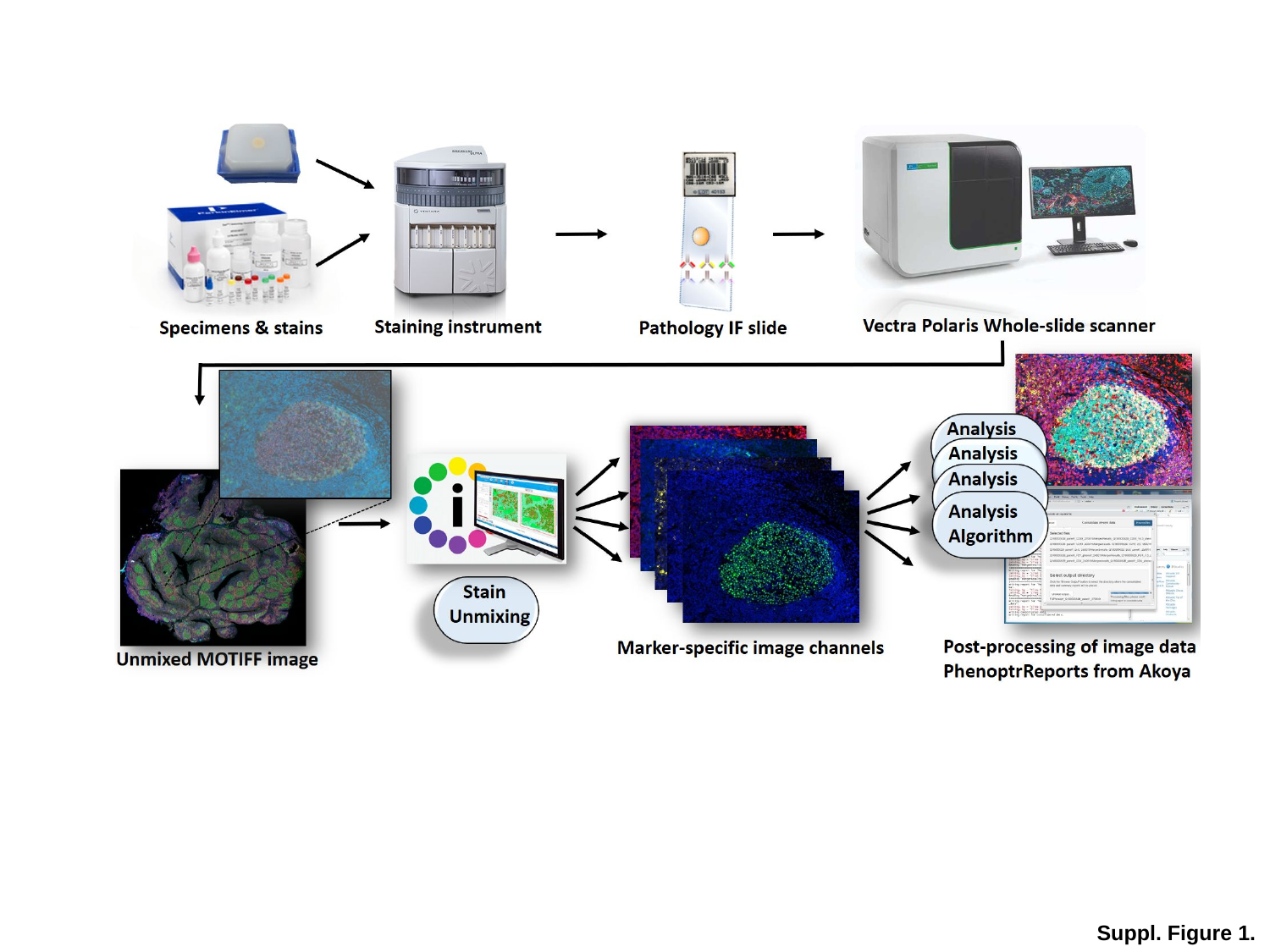

Suppl. Figure 1.

## Slide 2
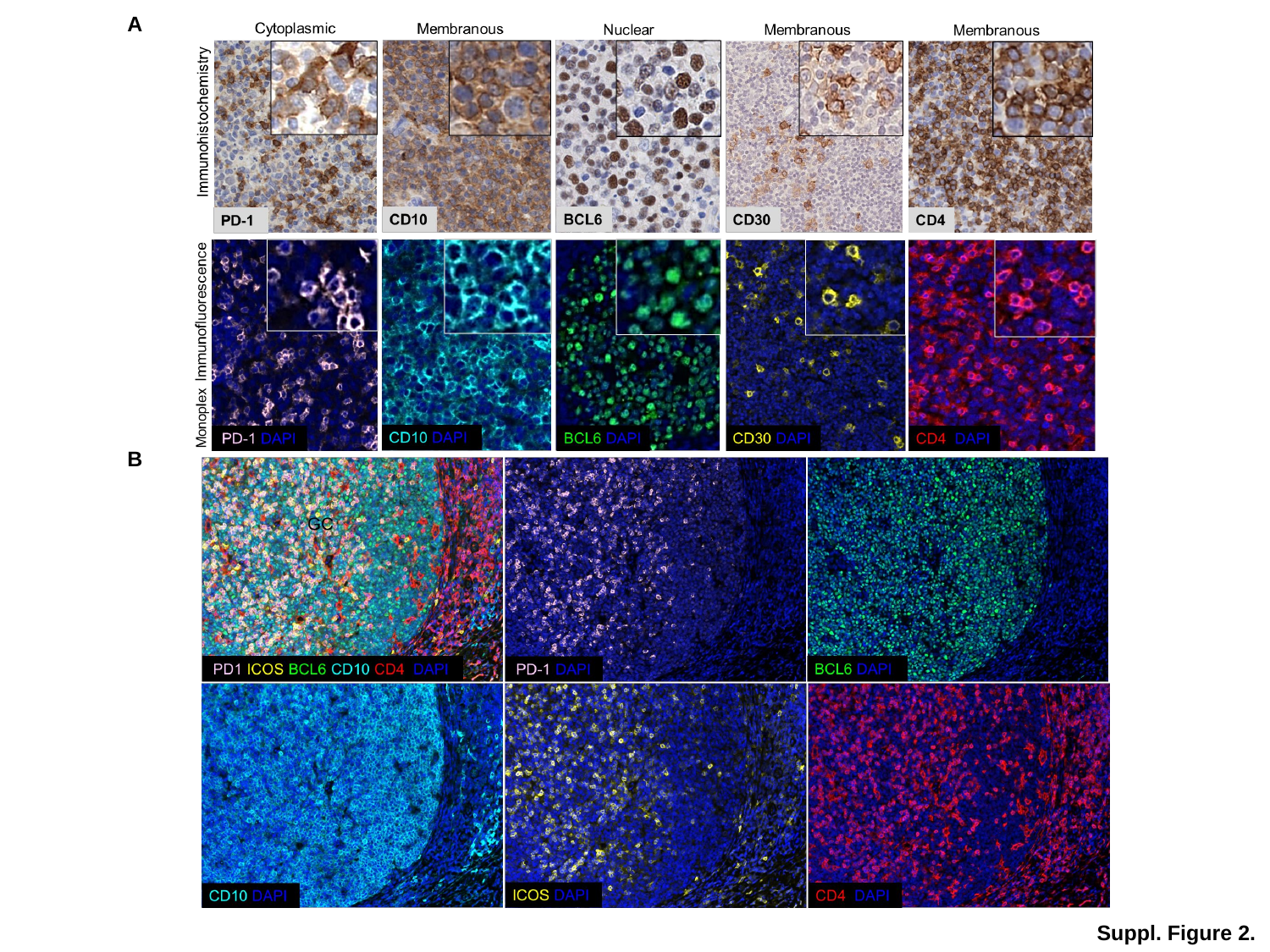

A
B
Suppl. Figure 2.

## Slide 3
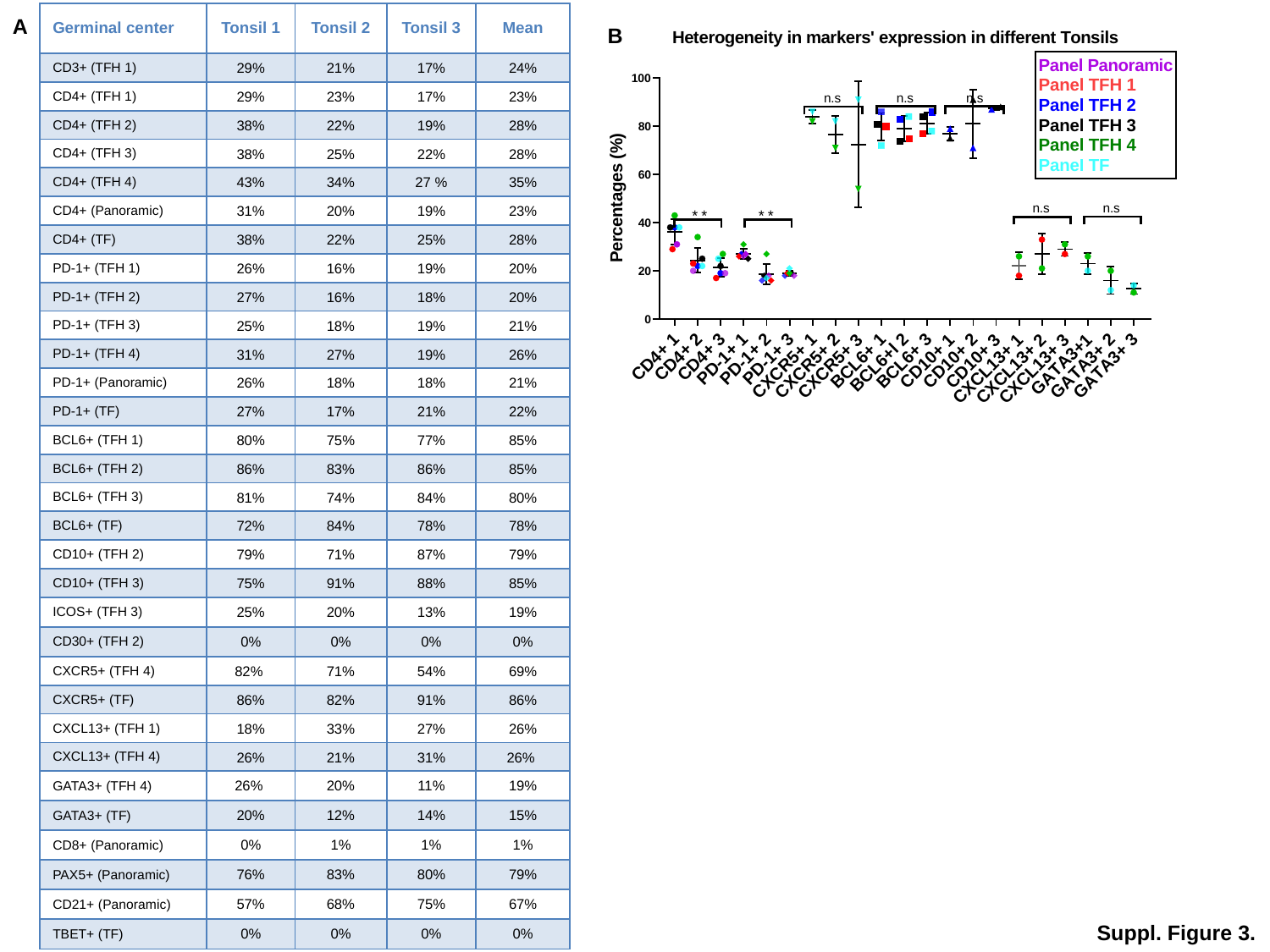

| Germinal center | Tonsil 1 | Tonsil 2 | Tonsil 3 | Mean |
| --- | --- | --- | --- | --- |
| CD3+ (TFH 1) | 29% | 21% | 17% | 24% |
| CD4+ (TFH 1) | 29% | 23% | 17% | 23% |
| CD4+ (TFH 2) | 38% | 22% | 19% | 28% |
| CD4+ (TFH 3) | 38% | 25% | 22% | 28% |
| CD4+ (TFH 4) | 43% | 34% | 27 % | 35% |
| CD4+ (Panoramic) | 31% | 20% | 19% | 23% |
| CD4+ (TF) | 38% | 22% | 25% | 28% |
| PD-1+ (TFH 1) | 26% | 16% | 19% | 20% |
| PD-1+ (TFH 2) | 27% | 16% | 18% | 20% |
| PD-1+ (TFH 3) | 25% | 18% | 19% | 21% |
| PD-1+ (TFH 4) | 31% | 27% | 19% | 26% |
| PD-1+ (Panoramic) | 26% | 18% | 18% | 21% |
| PD-1+ (TF) | 27% | 17% | 21% | 22% |
| BCL6+ (TFH 1) | 80% | 75% | 77% | 85% |
| BCL6+ (TFH 2) | 86% | 83% | 86% | 85% |
| BCL6+ (TFH 3) | 81% | 74% | 84% | 80% |
| BCL6+ (TF) | 72% | 84% | 78% | 78% |
| CD10+ (TFH 2) | 79% | 71% | 87% | 79% |
| CD10+ (TFH 3) | 75% | 91% | 88% | 85% |
| ICOS+ (TFH 3) | 25% | 20% | 13% | 19% |
| CD30+ (TFH 2) | 0% | 0% | 0% | 0% |
| CXCR5+ (TFH 4) | 82% | 71% | 54% | 69% |
| CXCR5+ (TF) | 86% | 82% | 91% | 86% |
| CXCL13+ (TFH 1) | 18% | 33% | 27% | 26% |
| CXCL13+ (TFH 4) | 26% | 21% | 31% | 26% |
| GATA3+ (TFH 4) | 26% | 20% | 11% | 19% |
| GATA3+ (TF) | 20% | 12% | 14% | 15% |
| CD8+ (Panoramic) | 0% | 1% | 1% | 1% |
| PAX5+ (Panoramic) | 76% | 83% | 80% | 79% |
| CD21+ (Panoramic) | 57% | 68% | 75% | 67% |
| TBET+ (TF) | 0% | 0% | 0% | 0% |
A
B
Suppl. Figure 3.

## Slide 4
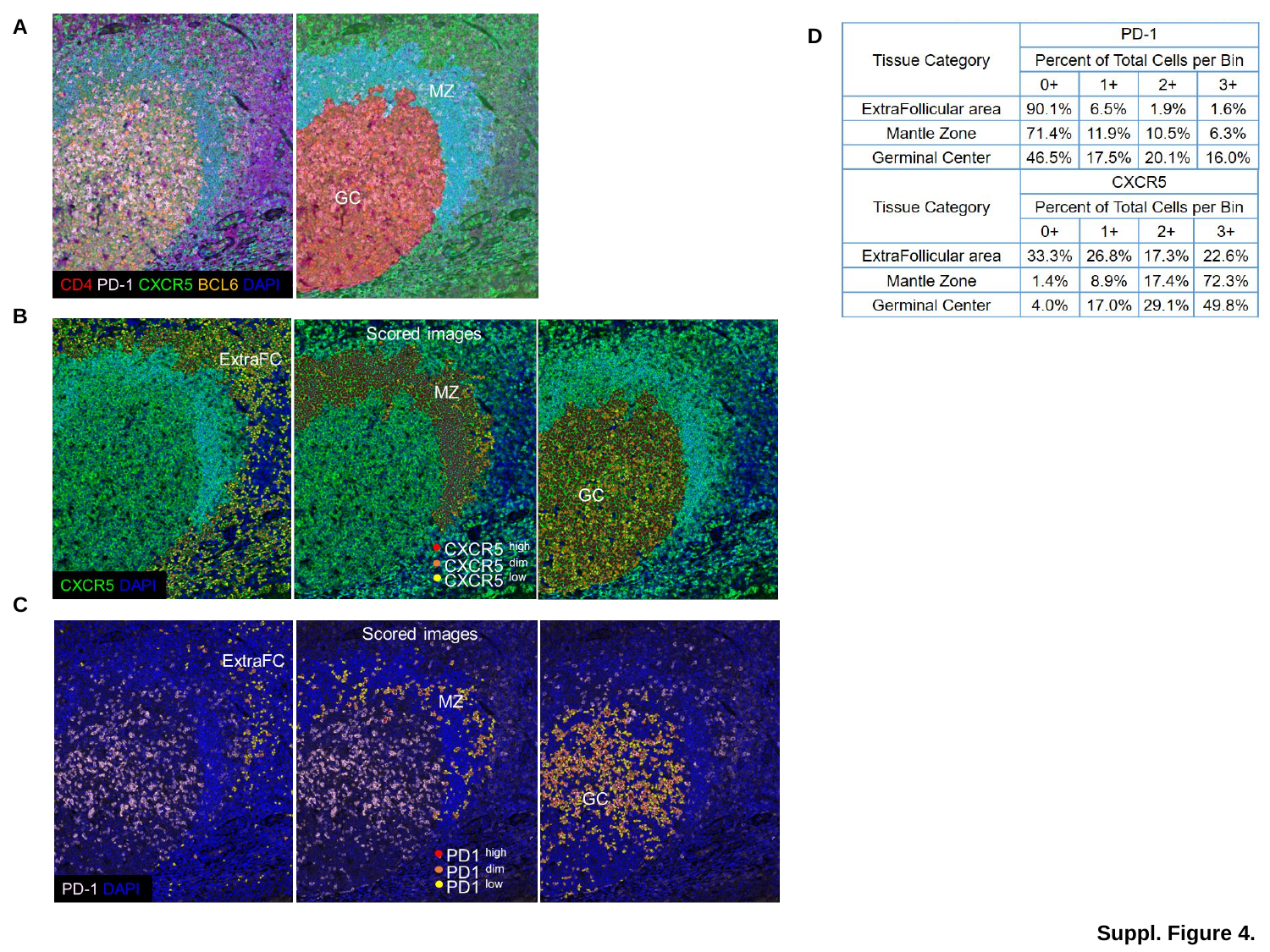

A
D
B
C
Suppl. Figure 4.

## Slide 5
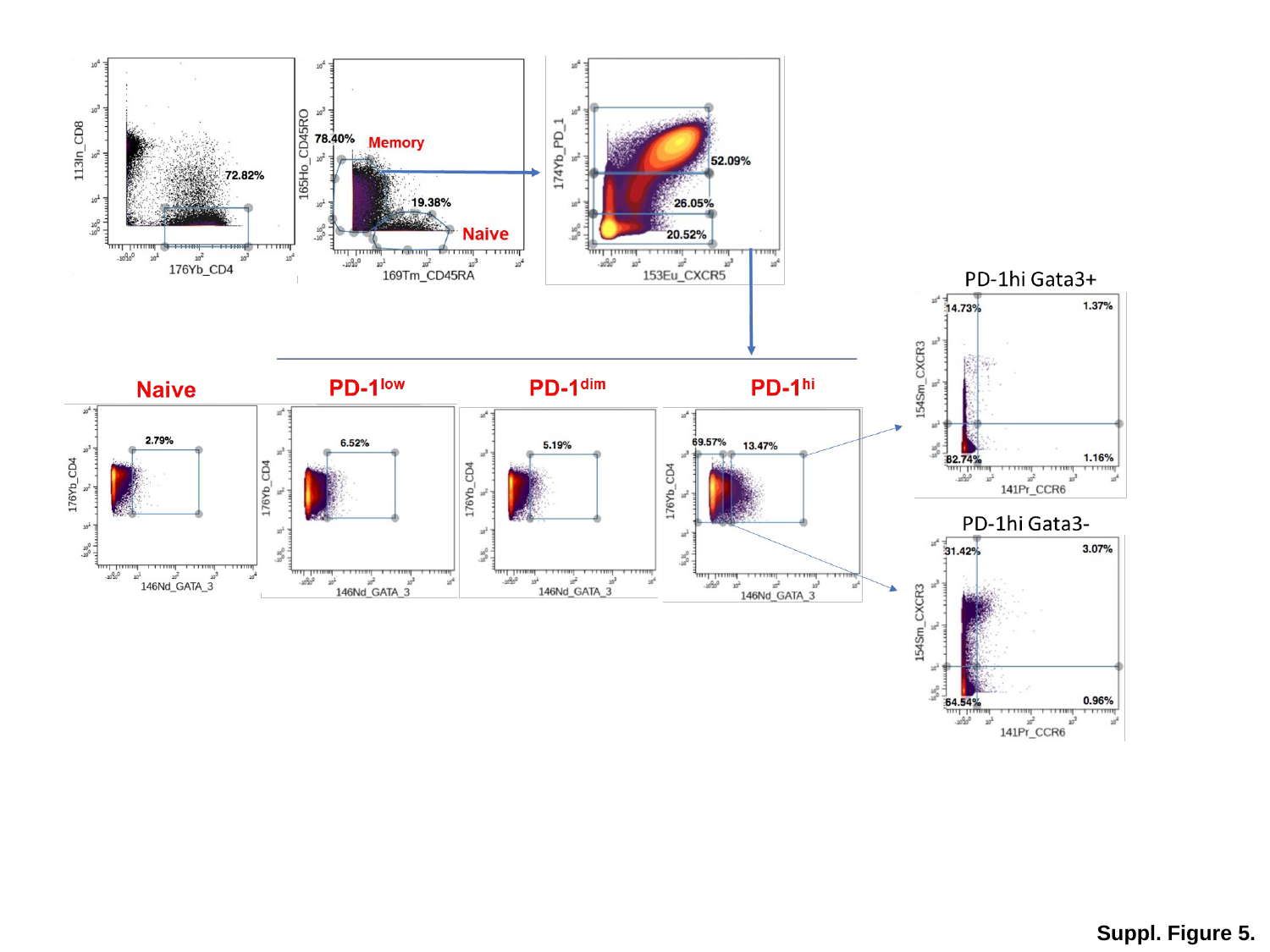

Suppl. Figure 5.

## Slide 6
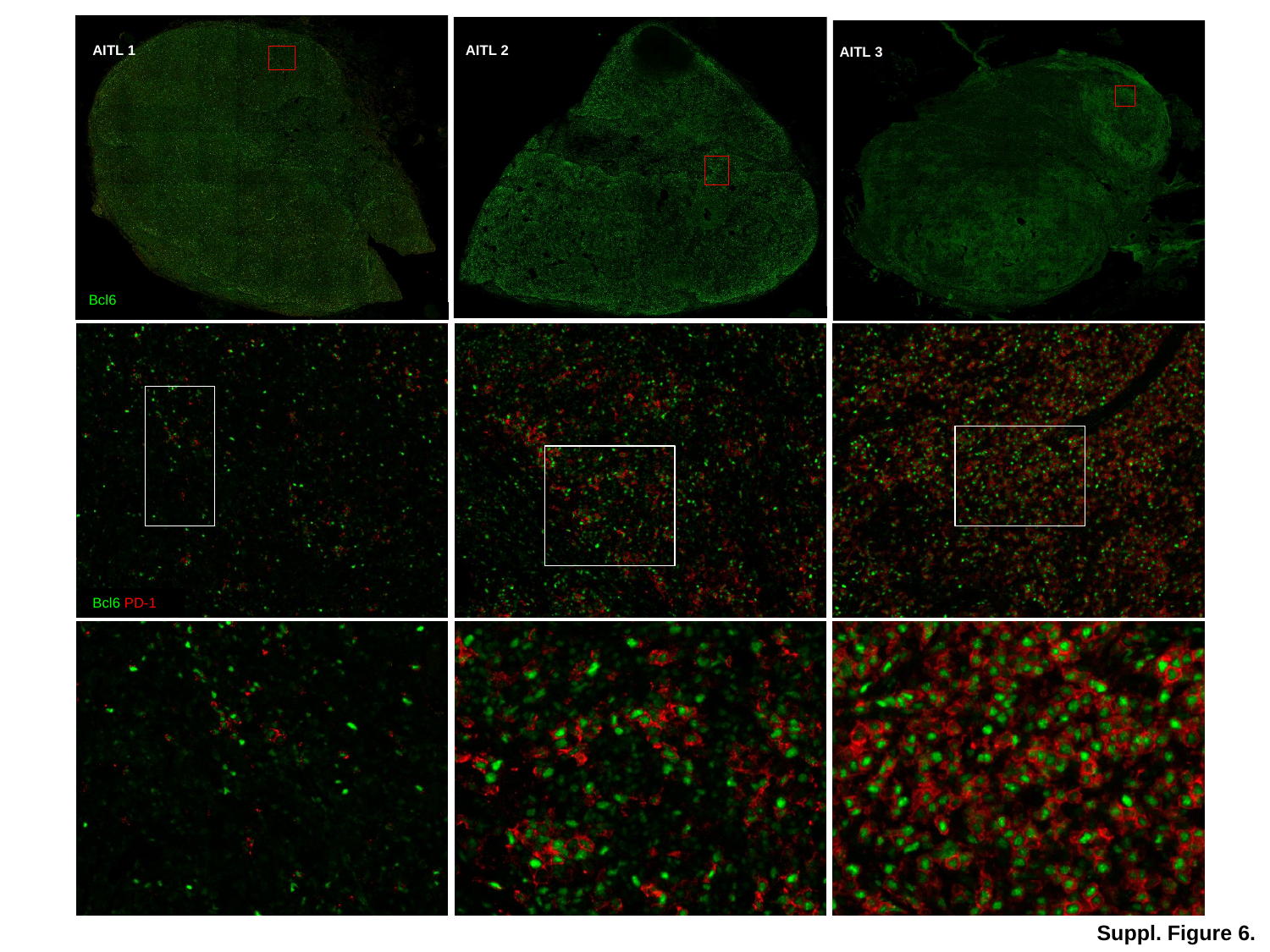

AITL 1
AITL 2
AITL 3
Bcl6
Bcl6 PD-1
Suppl. Figure 6.

## Slide 7
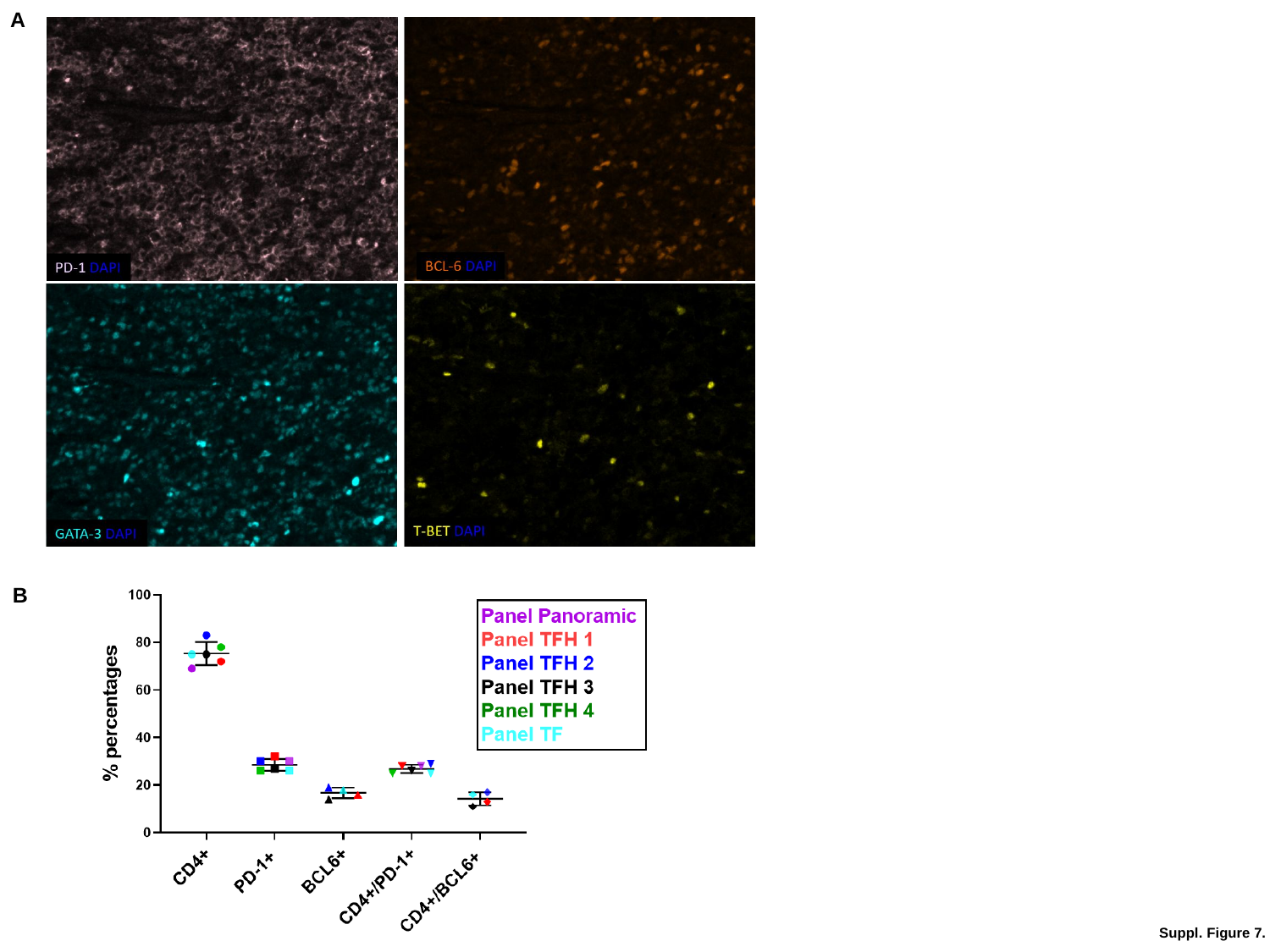

A
B
Suppl. Figure 7.

## Slide 8
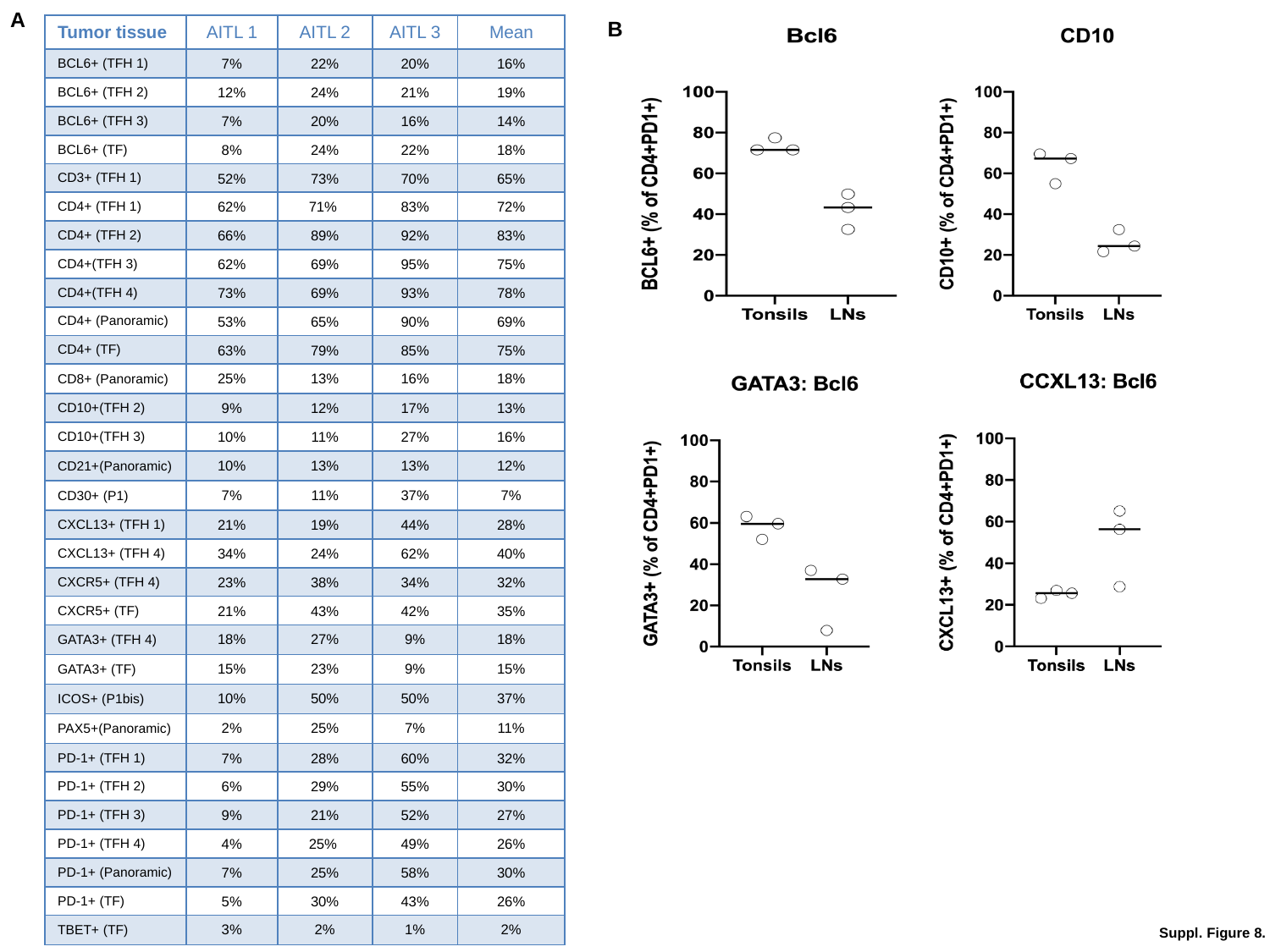

A
B
| Tumor tissue | AITL 1 | AITL 2 | AITL 3 | Mean |
| --- | --- | --- | --- | --- |
| BCL6+ (TFH 1) | 7% | 22% | 20% | 16% |
| BCL6+ (TFH 2) | 12% | 24% | 21% | 19% |
| BCL6+ (TFH 3) | 7% | 20% | 16% | 14% |
| BCL6+ (TF) | 8% | 24% | 22% | 18% |
| CD3+ (TFH 1) | 52% | 73% | 70% | 65% |
| CD4+ (TFH 1) | 62% | 71% | 83% | 72% |
| CD4+ (TFH 2) | 66% | 89% | 92% | 83% |
| CD4+(TFH 3) | 62% | 69% | 95% | 75% |
| CD4+(TFH 4) | 73% | 69% | 93% | 78% |
| CD4+ (Panoramic) | 53% | 65% | 90% | 69% |
| CD4+ (TF) | 63% | 79% | 85% | 75% |
| CD8+ (Panoramic) | 25% | 13% | 16% | 18% |
| CD10+(TFH 2) | 9% | 12% | 17% | 13% |
| CD10+(TFH 3) | 10% | 11% | 27% | 16% |
| CD21+(Panoramic) | 10% | 13% | 13% | 12% |
| CD30+ (P1) | 7% | 11% | 37% | 7% |
| CXCL13+ (TFH 1) | 21% | 19% | 44% | 28% |
| CXCL13+ (TFH 4) | 34% | 24% | 62% | 40% |
| CXCR5+ (TFH 4) | 23% | 38% | 34% | 32% |
| CXCR5+ (TF) | 21% | 43% | 42% | 35% |
| GATA3+ (TFH 4) | 18% | 27% | 9% | 18% |
| GATA3+ (TF) | 15% | 23% | 9% | 15% |
| ICOS+ (P1bis) | 10% | 50% | 50% | 37% |
| PAX5+(Panoramic) | 2% | 25% | 7% | 11% |
| PD-1+ (TFH 1) | 7% | 28% | 60% | 32% |
| PD-1+ (TFH 2) | 6% | 29% | 55% | 30% |
| PD-1+ (TFH 3) | 9% | 21% | 52% | 27% |
| PD-1+ (TFH 4) | 4% | 25% | 49% | 26% |
| PD-1+ (Panoramic) | 7% | 25% | 58% | 30% |
| PD-1+ (TF) | 5% | 30% | 43% | 26% |
| TBET+ (TF) | 3% | 2% | 1% | 2% |
Suppl. Figure 8.
